# Supplementary material for: Opsin 3 mediates UVA-induced keratinocyte supranuclear melanin cap formation
Source: Commun Biol. 2023 Mar 3;6:238. doi: 10.1038/s42003-023-04621-8 (PMC9984416; doi:10.1038/s42003-023-04621-8)

Fig. s1: UVA induces melanin cap formation in keratinocytes.

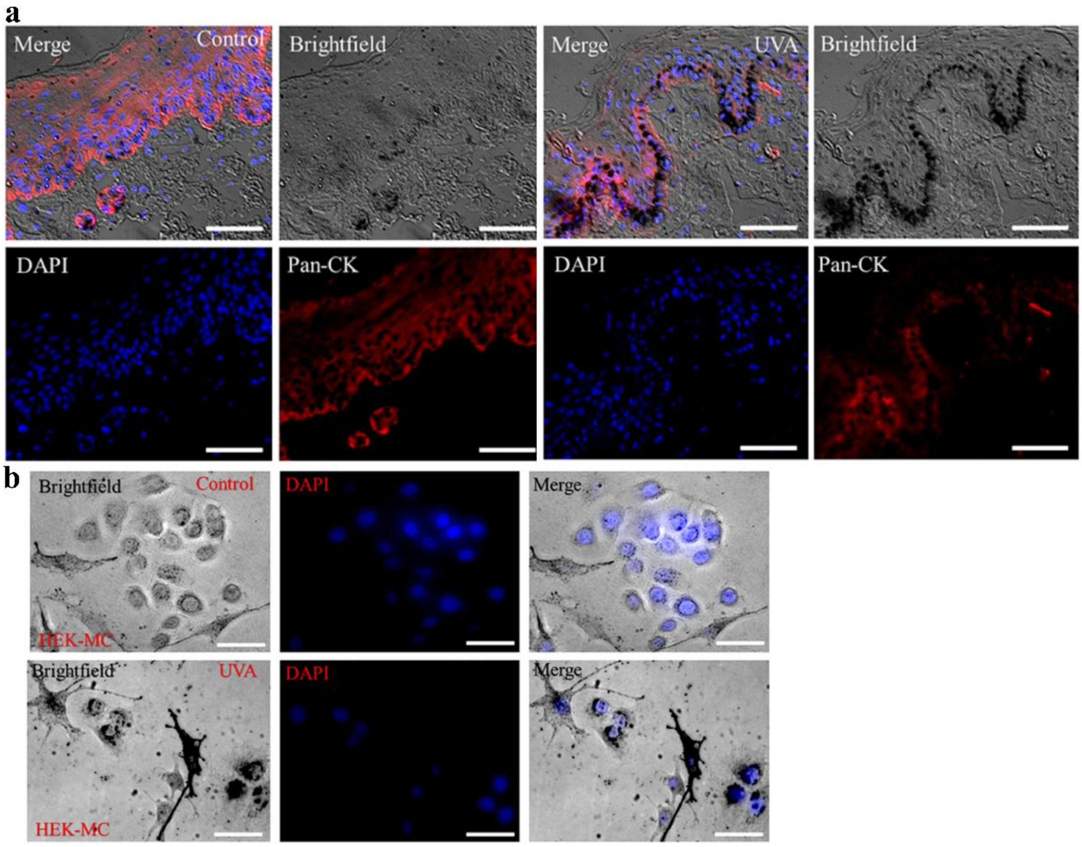

a Masson-Fontana (MF) staining shows keratinocytes supranuclear melanin cap localization after 48 h culture of control (top) and UVA-treated(bottom) skin explant. pan-Cytokeratin (Pan-CK) (red) as a marker of keratinocytes. Nuclei were counterstained with DAPI. Images were and analyzed by brightfield/fluorescence microscopy. scale bar = 20 μm. **b** MC co-culture with HaCaT was irradiated by UVA, MF staining demonstrated keratinocytes supranuclear melanin cap formation. Nuclei were counterstained with DAPI. Images were analyzed by brightfield/fluorescence microscopy. scale bar = 20 μm.

Fig. s2: Melanin particles from melanocytes are fed into HaCaT after UVA irradiation.

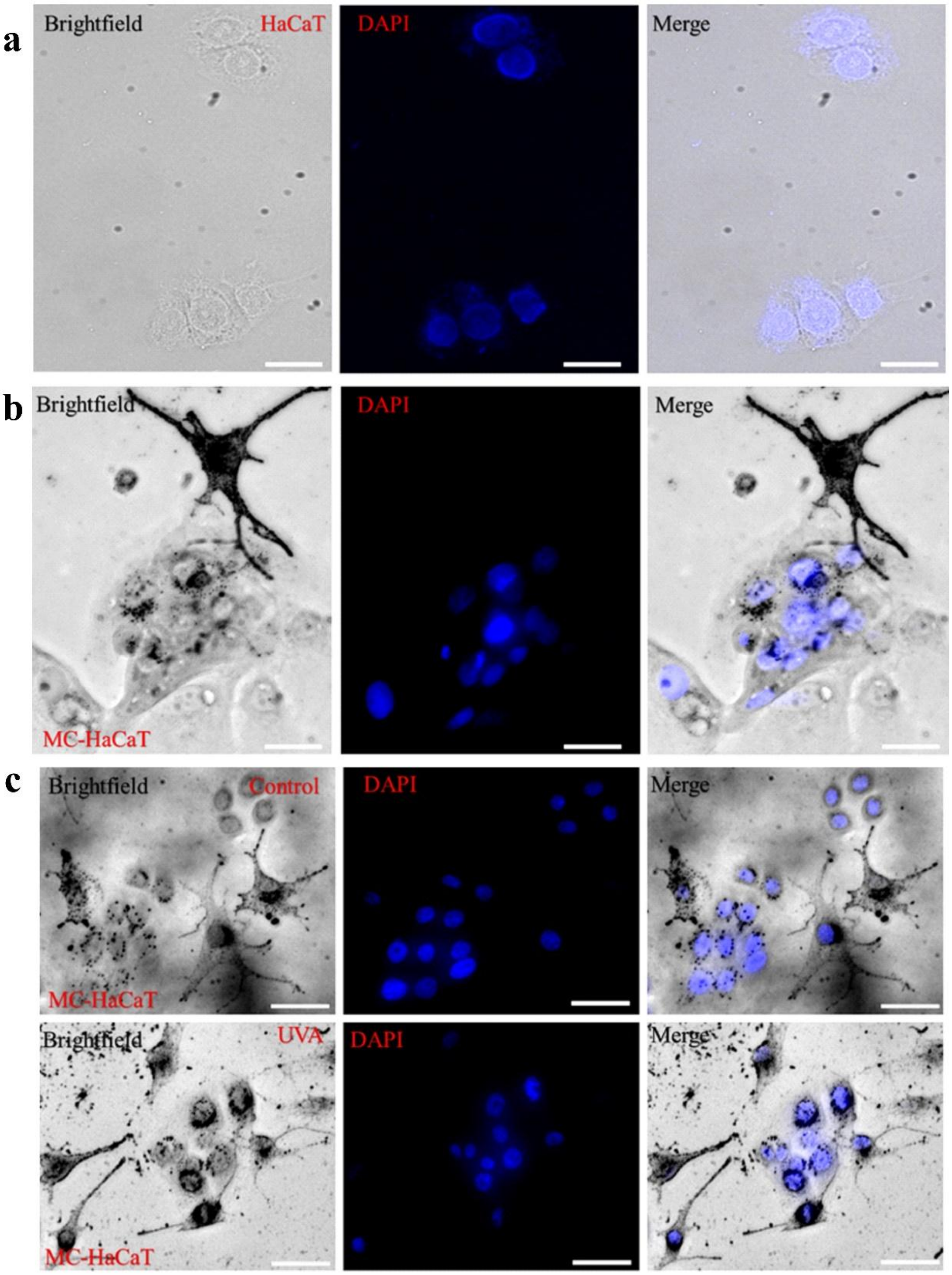

a MF staining showed melanin in HaCaT. Nuclei were counterstained with DAPI. Cells were fixed and analyzed by brightfield/fluorescence microscopy. scale bar = 20 μm. **b** After melanin was fed to HaCaT by MC, Masson-Fontana staining was used to detect melanin in HaCaT. Nuclei were counterstained with DAPI. Images were

analyzed by brightfield/fluorescence microscopy. scale bar = 20 μm. **c** MC co-culture with HaCaT was irradiated by UVA, MF staining demonstrated melanin cap formation. Nuclei were counterstained with DAPI. Images were analyzed by brightfield/fluorescence microscopy. scale bar = 20 μm.

Fig. s3: UVA increases Dync1i1 and DCTN1 expression in keratinocytes.

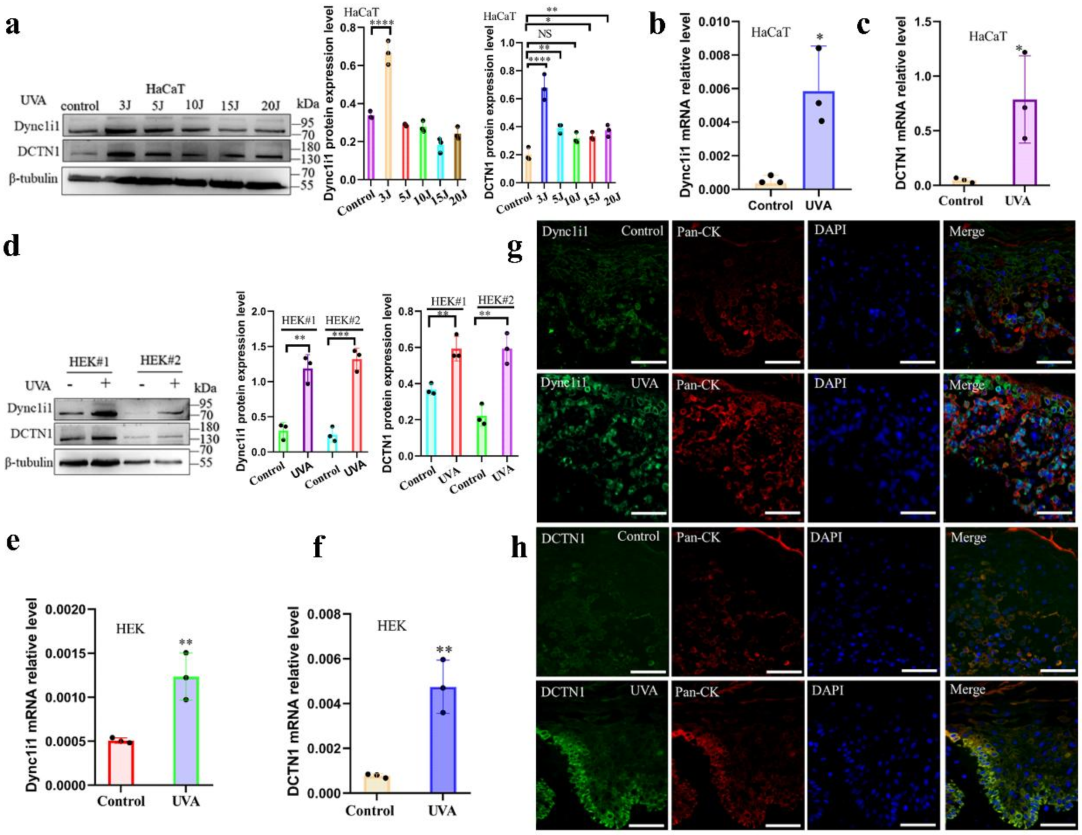

a Relative Dync1i1 and DCTN1 protein expression level was shown after exposure to UVA radiation (0 J cm^{-2}, 3 J cm^{-2}, 5 J cm^{-2}, 10 J cm^{-2}, 15 J cm^{-2}, 20 J cm^{-2}). WB analyses were normalized using β-tubulin as a loading control and the relative protein level was quantified using Quantity One software. n =3 independent experiments. Statistical significance was determined by one-ANOVA with post-test. *P<0.05, **P<0.01, ****P<0.0001. **b and c** After UVA irradiation, RT-qPCR was used to analyze changes of Dync1i1 and DCTN1 mRNA expression levels in HaCaT. The mRNA levels were normalized to GAPDH levels (n=3 independent experiments). Statistical significance was determined by Student's t test analysis. *P<0.05. **d** After UVA irradiation, WB was used to analyze changes of Dync1i1 and DCTN1 protein expression levels in HEK. The relative protein level was quantified using Quantity One software. n=3 independent experiments. Statistical significance was determined by t-test analysis. **P<0.01, ***P<0.001. **e and f** After UVA irradiation, RT-qPCR was used to analyze changes of Dync1i1 and DCTN1 expression in HEK. The mRNA levels were normalized to GAPDH levels (n =3 independent experiments). Statistical

significance was determined by t-test analysis. **P<0.01. **g and h** Dync1i1 or DCTN1 expression (green) colocalized with HEK marker Pan-CK (red) in skin explant with immunofluorescence staining, without UVA (top) or with UVA (bottom). The nucleus was restrained with DAPI. n=3 independent experiments. scale bar = 20 μm.

Fig. s4: UVA mediates melanin cap formation through DCTN1.

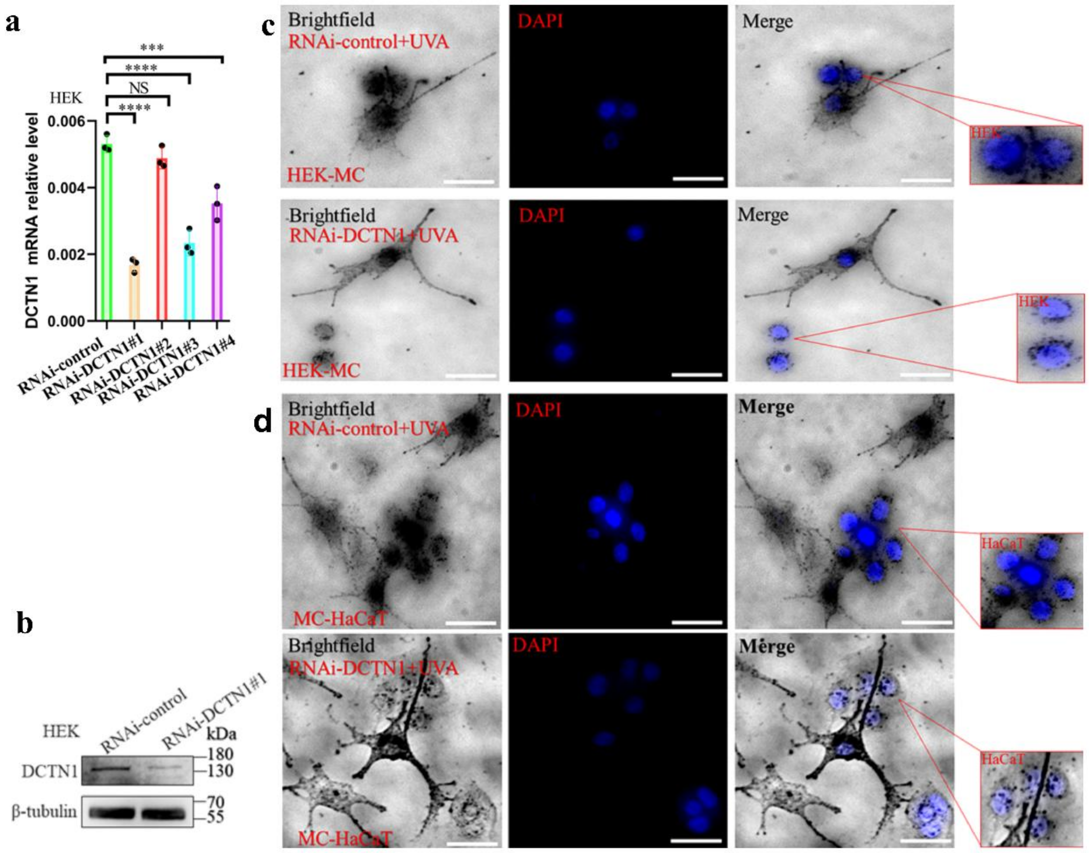

a DCTN1 mRNA levels in HEK expressing control or DCTN1-targeting siRNA (DCTN1#1, DCTN1#2, DCTN1#3 and DCTN1#4). The mRNA expression level of DCTN1 after targeting siRNA of DCTN1 in HEK (DCTN1#1, DCTN1#2, DCTN1#3 and DCTN1#4). mRNA levels of DCTN1 relative to GAPDH were analyzed by RT-qPCR. RNAi-DCTN1#1 HEK have decreased DCTN1 mRNA levels by ~75%, compared with RNAi-control transduced HEK (n =3 independent experiments). Statistical significance was determined by one-ANOVA with post-test. ****P*<0.001, *****P*<0.0001. **b** WB analysis of DCTN1 relative to β-tubulin protein levels was performed. The relative protein level was quantified using Quantity One software. RNAi-DCTN1#1 HEK have decreased DCTN1 protein levels by ~75%, compared with RNAi-control transduced HEK. **c** After UVA irradiation of control group and siDCTN1 group in co-culture of MC and HEK, MF staining demonstrated melanin cap formation. Nuclei were counterstained with DAPI. Images were analyzed by brightfield/fluorescence microscopy. scale bar = 20 μm. **d** After UVA irradiation of

control group and siDCTN1 group in co-culture of MC and HaCaT, MF staining demonstrated melanin cap formation. Nuclei were counterstained with DAPI. Images were analyzed by brightfield/fluorescence microscopy. scale bar = 20 μm.

Fig. s5: 3J/cm^2 UVA does not cause the production of reactive oxygen species in keratinocytes.

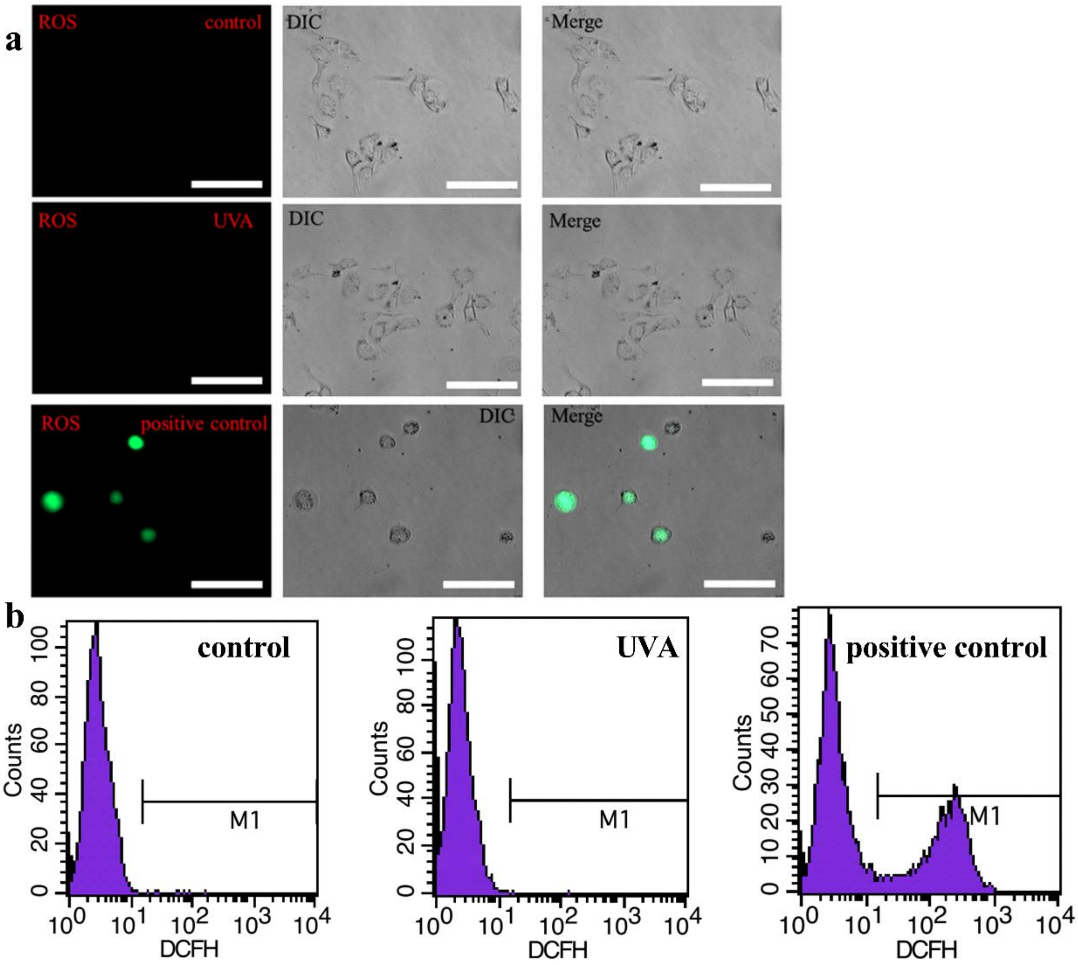

a Fluorescence microscope observation of ROS in keratinocytes. scale bar = 20 μm. **b** Analysis of ROS in keratinocytes by flow cytometry. n =3 independent experiments.

Fig. s6: After using small interference technology to inhibit OPN3, the ability of UVA to induce the formation of melanin cap decreased.

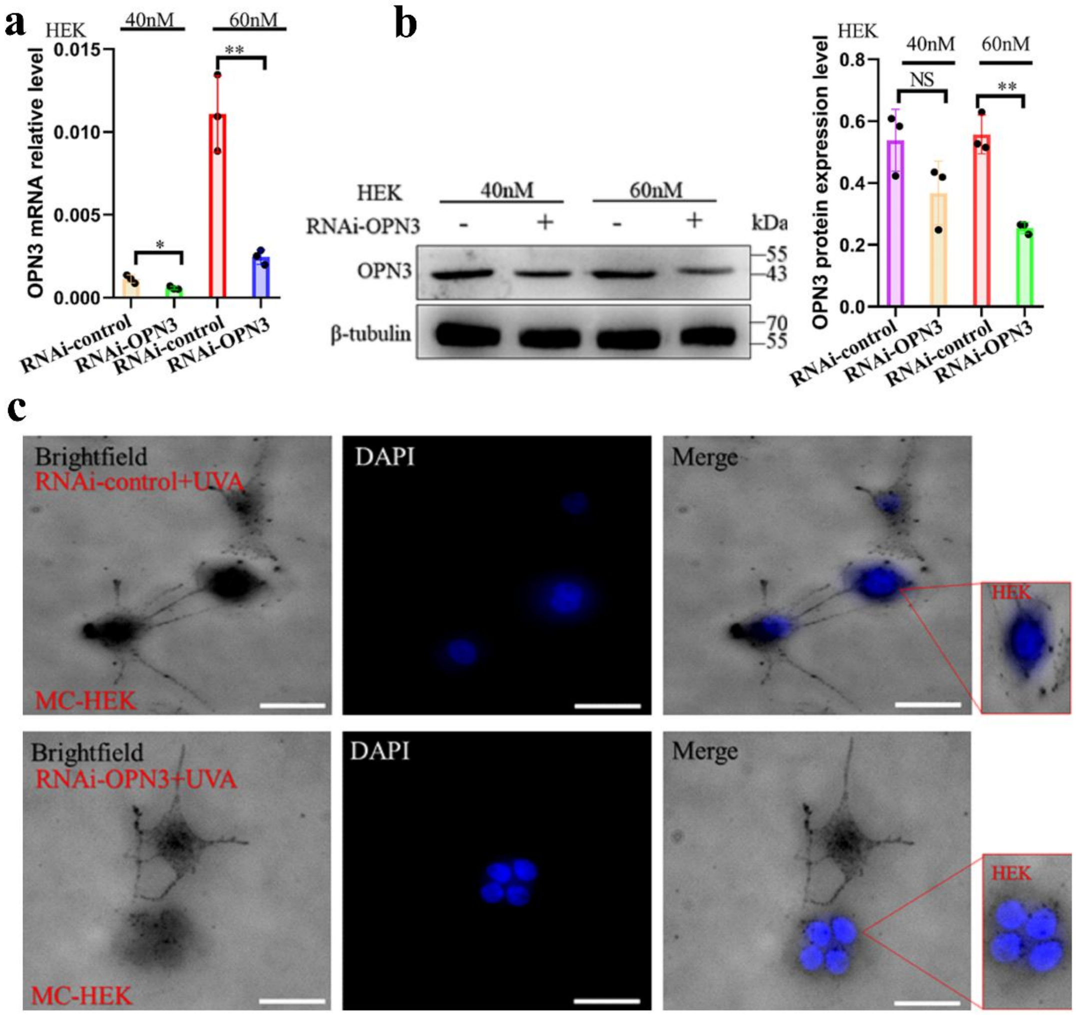

a OPN3 mRNA levels in HEK expressing control or OPN3-targeting siRNA. RT-qPCR analysis of OPN3 relative to GAPDH mRNA levels was performed. n = 3 independent experiments. Statistical significance was determined by Student's t test analysis. *P<0.05; **P<0.01. **b** HEK were transfected with siRNA directed against OPN3 or control. The efficiency of siRNA was determined by WB analysis (n =3 independent experiments). WB analyses were normalized using β-tubulin as a loading control and the relative protein level was quantified using Quantity One software. Statistical significance was determined by Student's t test analysis. **P<0.01. **c** After UVA irradiation of control group and siOPN3 group in co-culture of MC and HEK, MF staining demonstrated melanin cap formation. Nuclei were counterstained with

DAPI. Images were analyzed by brightfield/fluorescence microscopy. scale bar = 20.

Fig.s7: After inhibiting OPN3 by lentivirus transfection technology, the ability of UVA to induce the formation of melanin cap decreased.

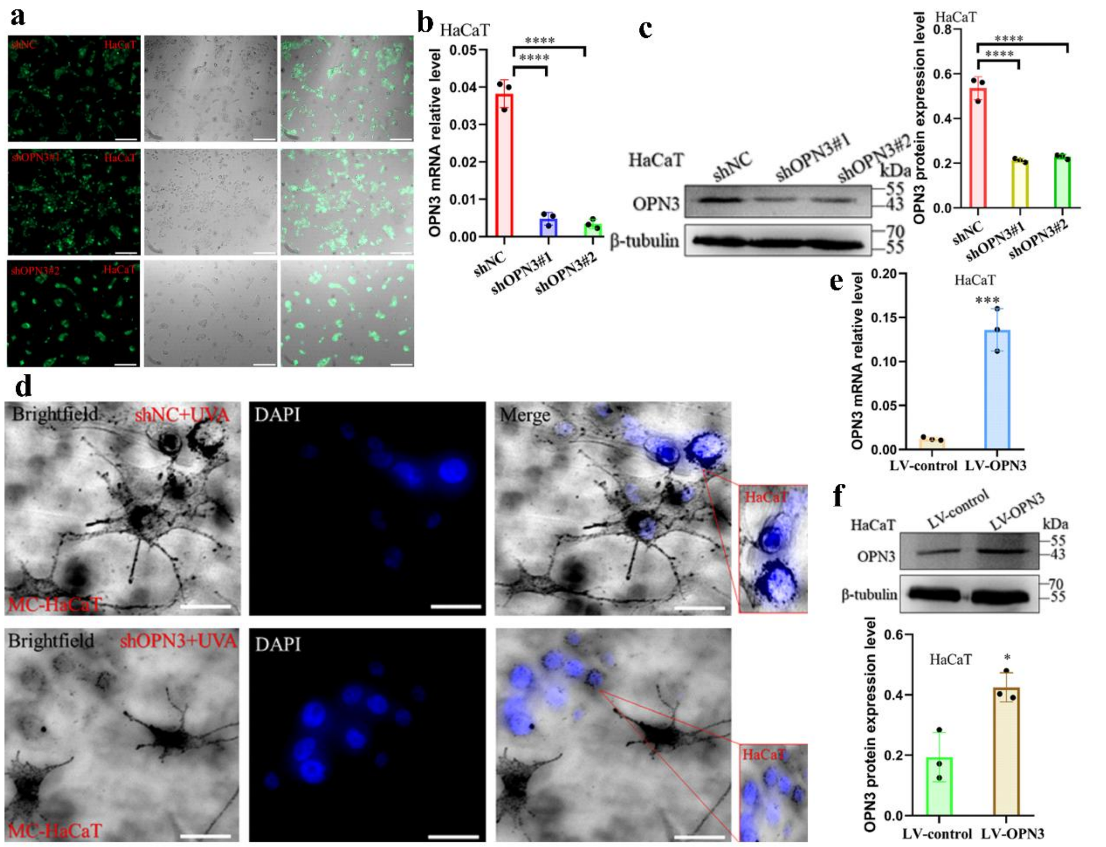

a HaCaT was transfected with lentivirus shOPN3 (shOPN3 # 1 or shOPN3 # 2) and control lentivirus (shNC). Observe the fluorescence of shOPN3 and shNC under fluorescence microscope. scale bars = 50 μm. **b** RT-qPCR was used to analyze transfection efficiency of the OPN3 mRNA expression levels. n =3 independent experiments. Statistical significance was determined by one-ANOVA with post-test. *****P*<0.0001. **c** WB were used to analyze transfection efficiency of the OPN3 protein levels. β-tubulin was used as a loading control. The relative protein level was quantified using Quantity One software. n =3 independent experiments. Statistical significance was determined by Student's t test analysis. *****P*<0.0001. **d** After UVA irradiation of shNC group and shOPN3 group, MF staining demonstrated melanin cap formation. The nucleus was restrained with DAPI. Cells were fixed and analyzed by brightfield/fluorescence microscopy. scale bar = 20 μm. **e** HaCaT was transfected with lentivirus overexpression OPN3 (LV-OPN3) and control lentivirus (LV-control). RT-

qPCR was used to analyze transfection efficiency of the OPN3 mRNA expression levels. n =3 independent experiments. Statistical significance was determined by Student's t test analysis. ***P<0.001. **f** WB were used to analyze transfection efficiency of the OPN3 protein levels. β-tubulin was used as a loading control. The relative protein level was quantified using Quantity One software. n =3 independent experiments. Statistical significance was determined by Student's t test analysis. *P<0.05.

Fig. s8: The intracellular calcium level was detected through fluorometric Ca^{2+} imaging.

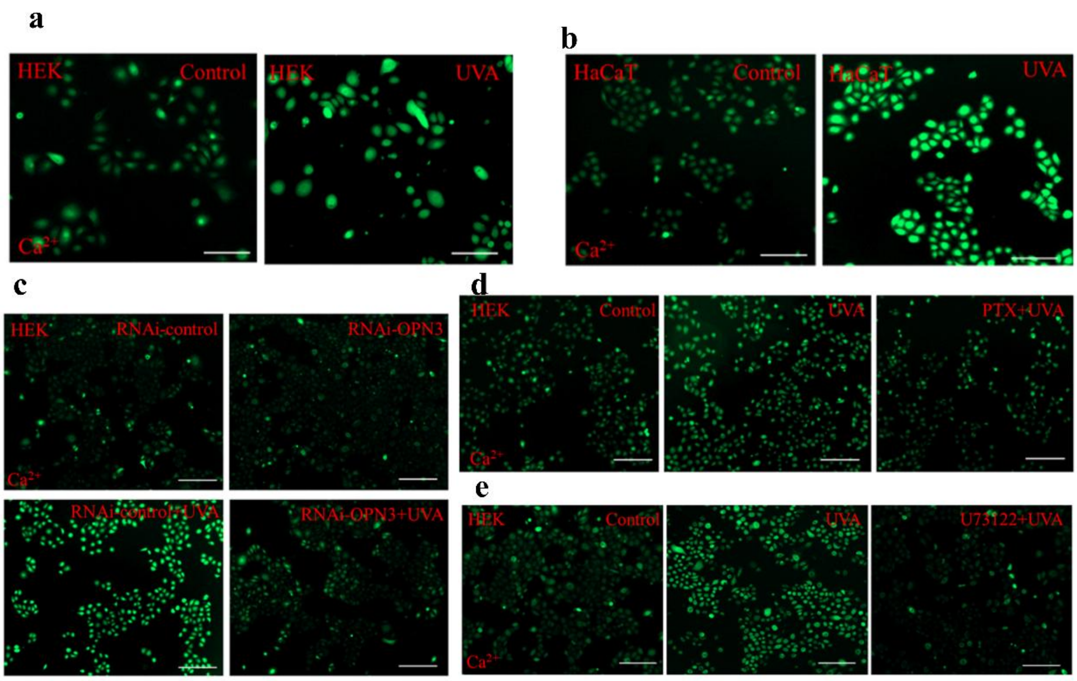

a and b Images of representative HEK and HaCaT loaded with the Ca^{2+} indicator Fluo-3 and irradiated with UVA. scale bar = 50 μm. **c** After siRNA inhibited OPN3 irradiated without or with UVA, the fluorescence intensity of Ca^{2+} was observed by fluorescence microscope. Scale bar = 50 μm. **d** Images of representative HEK loaded with the Ca^{2+} indicator Fluo-3 and treatment with PTX were stimulated with 3 J cm^{-2} UVA. scale bar = 50 μm. **e** Images of representative HEK loaded with the Ca^{2+} indicator Fluo-3 and treatment with U73122 were stimulated with 3 J cm^{-2} UVA. scale bar = 50 μm.

Fig. s9: PTX, U73122 and MK-2206 affect cell viability.

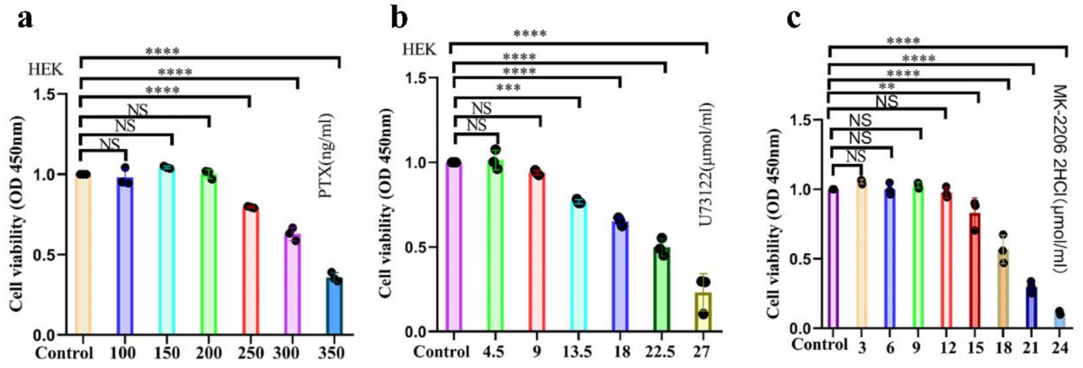

a CCK8 analysis of cell viability in different concentrations of PTX. n =3 independent experiments. Statistical significance was determined by one-ANOVA with post-test. * ****P<0.0001. **b** CCK8 analysis of cell viability in different concentrations of U73122. n =3 independent experiments. Statistical significance was determined by one-ANOVA with post-test. ***P<0.001, ****P<0.0001. **c** CCK8 analysis of cell viability in different concentrations of MK-2206. n =3 independent experiments. Statistical significance was determined by one-ANOVA with post-test. **P<0.01, ****P<0.0001.

Fig. s10: Source blots for western blot.

Relative to Fig.2c

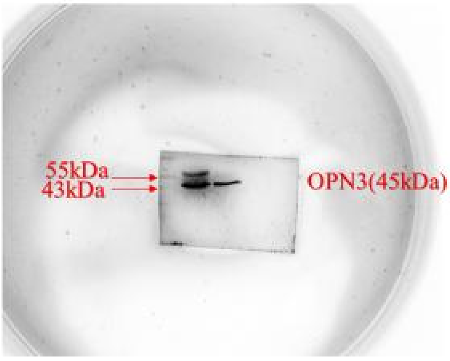

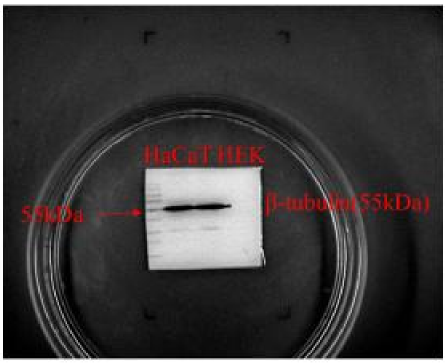

Relative to Fig.2f

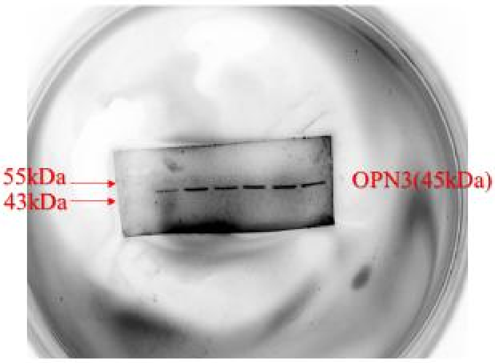

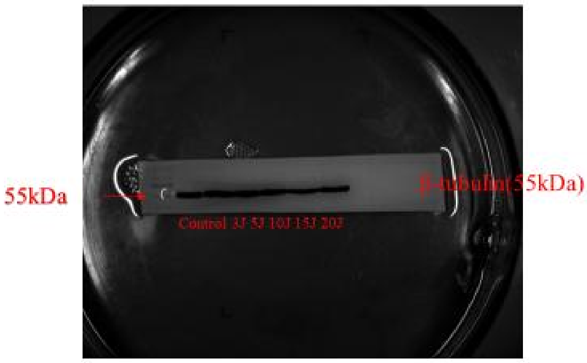

Relative to Fig.2h

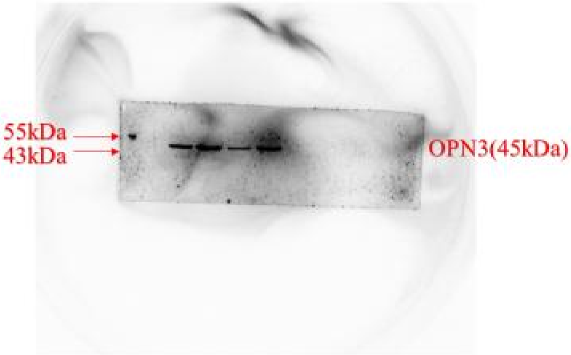

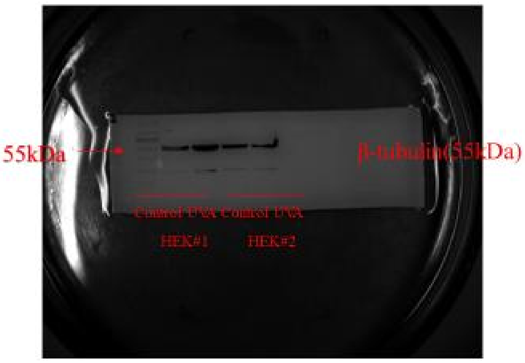

Relative to Fig.3b

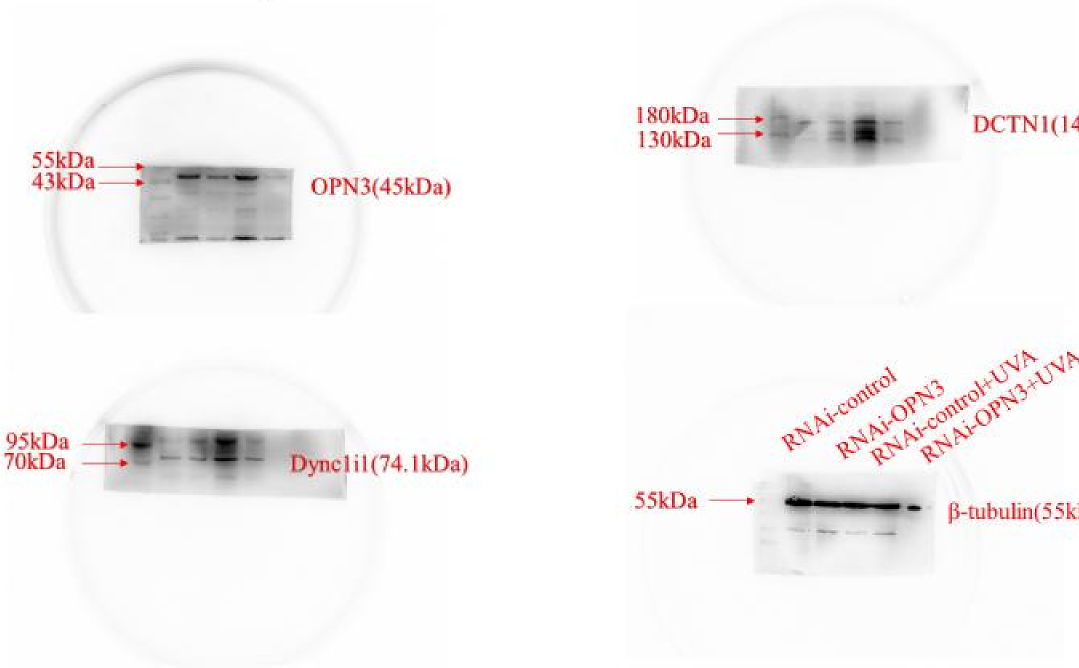

Relative to Fig.4a

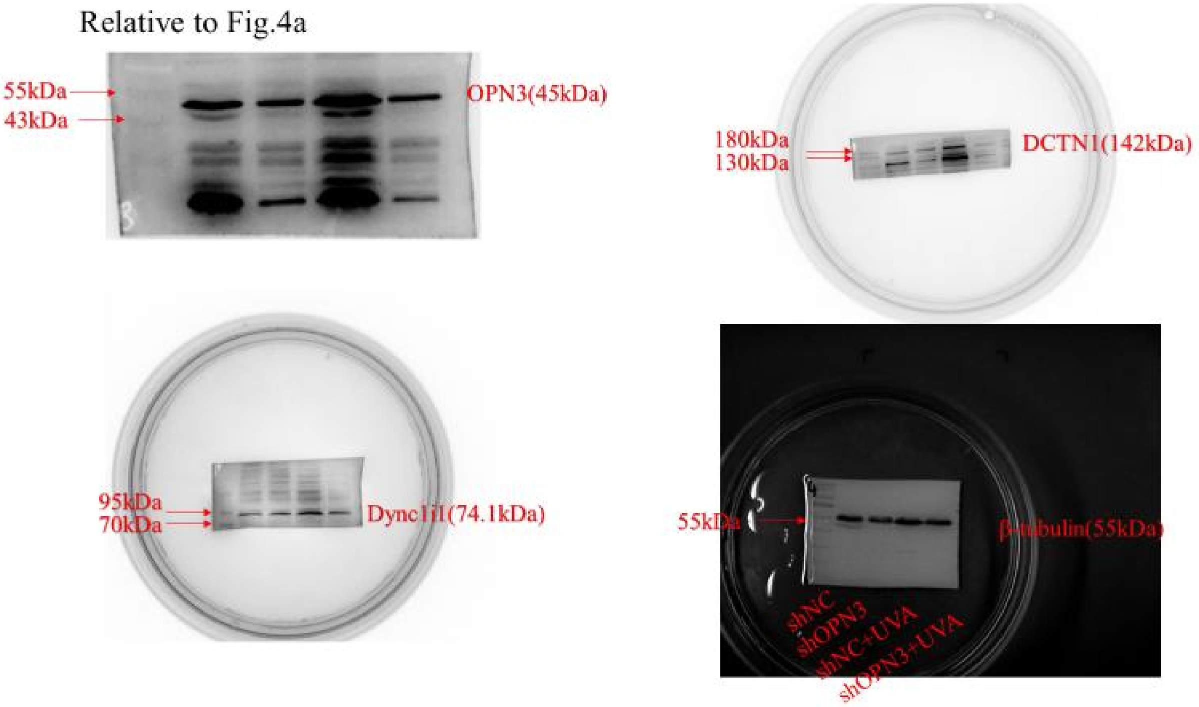

Relative to Fig.4e

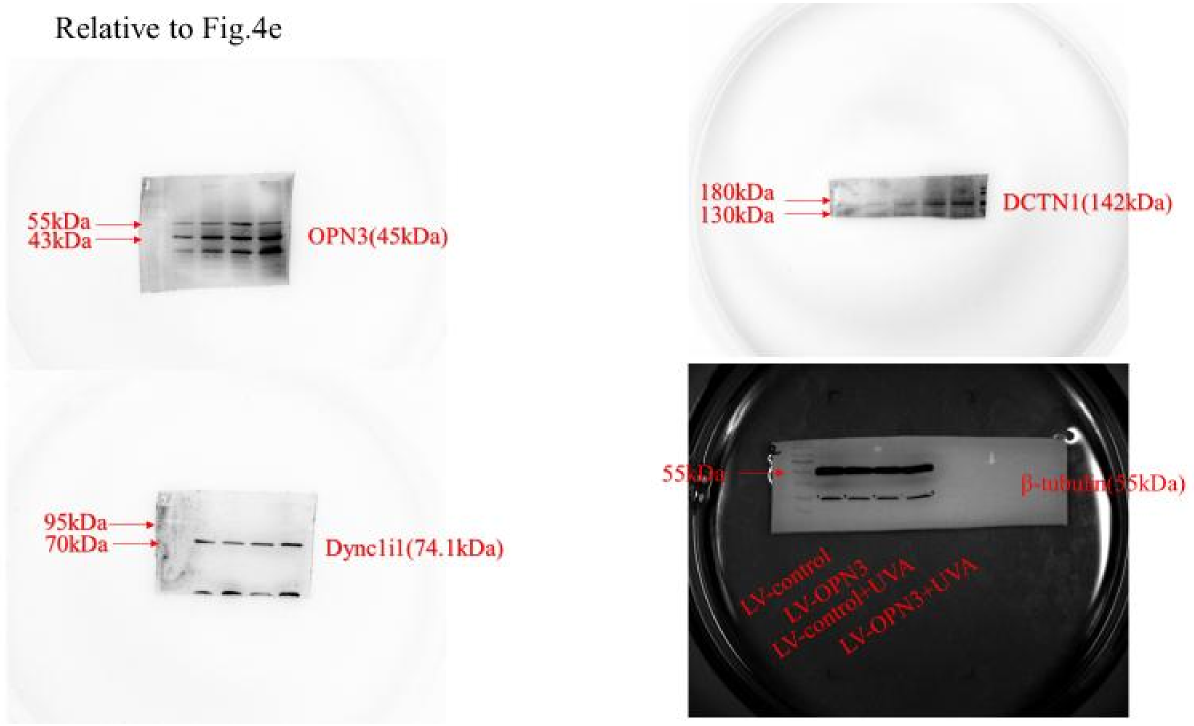

Relative to Fig.5c

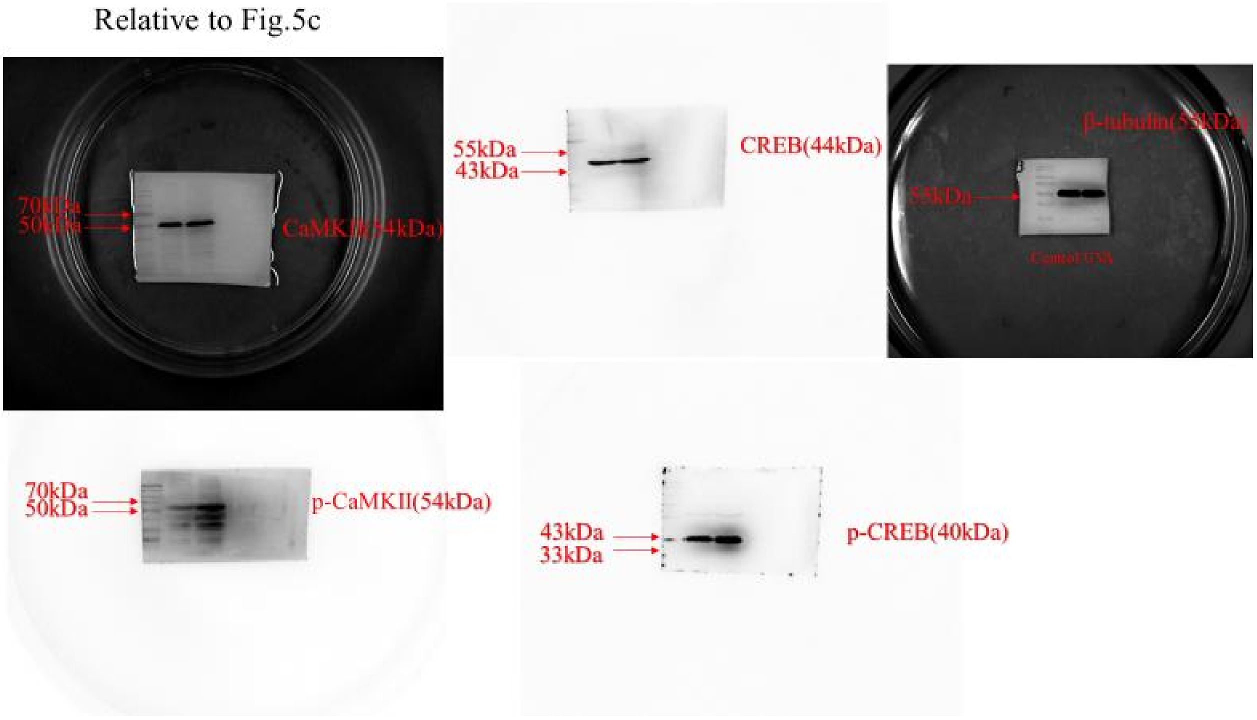

Relative to Fig.5d

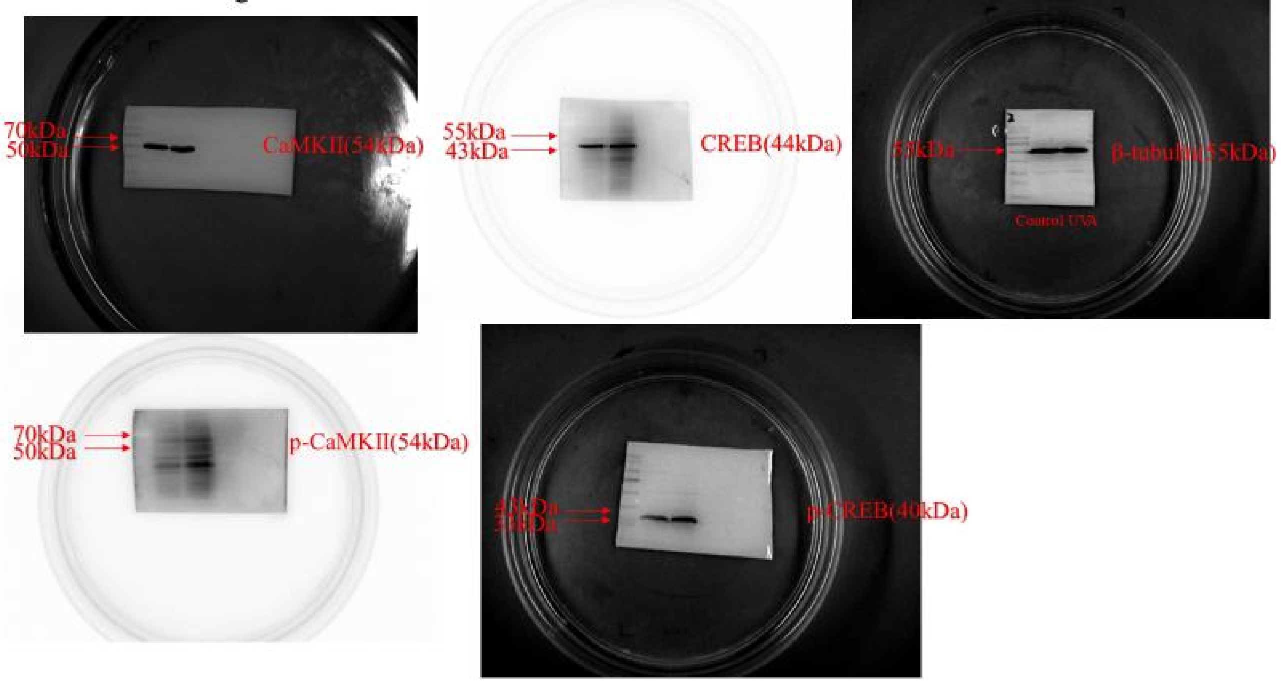

Relative to Fig.5f

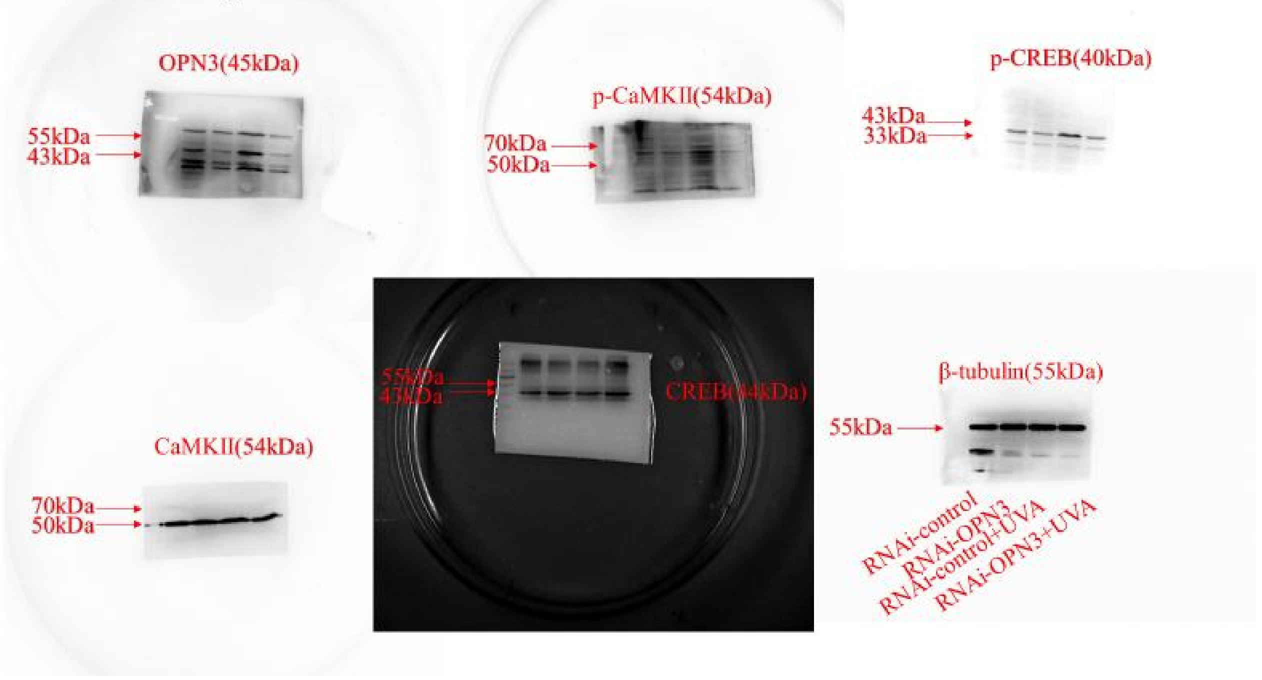

Relative to Fig.6b

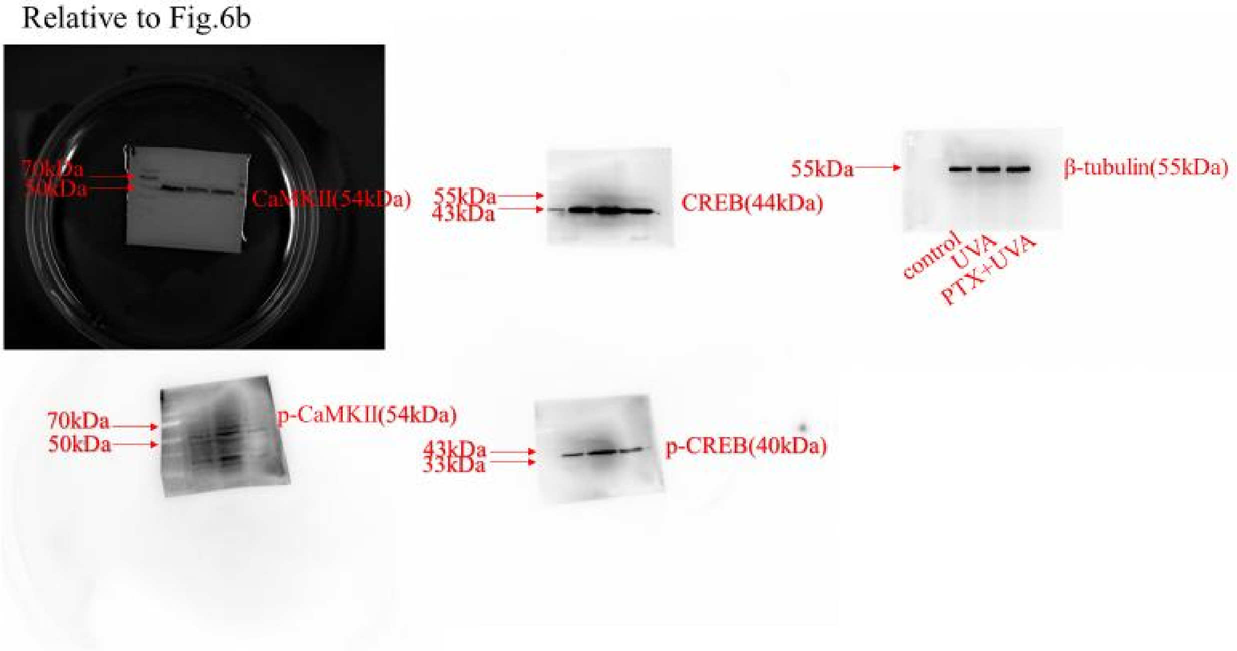

Relative to Fig.6c

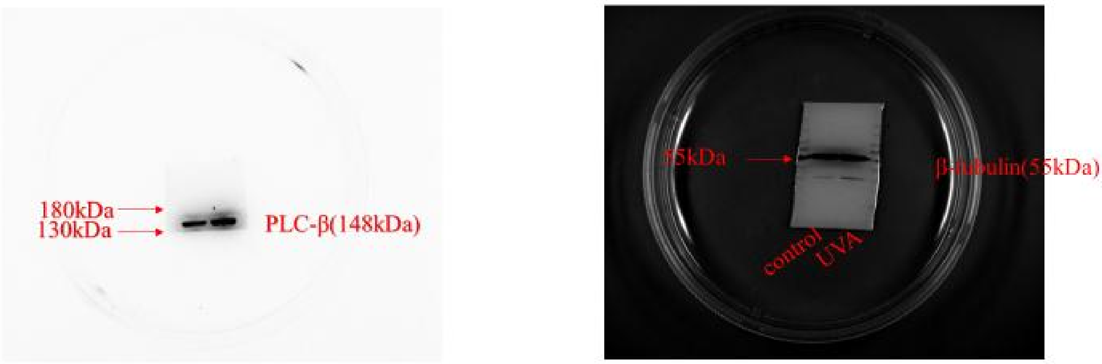

Relative to Fig.6d

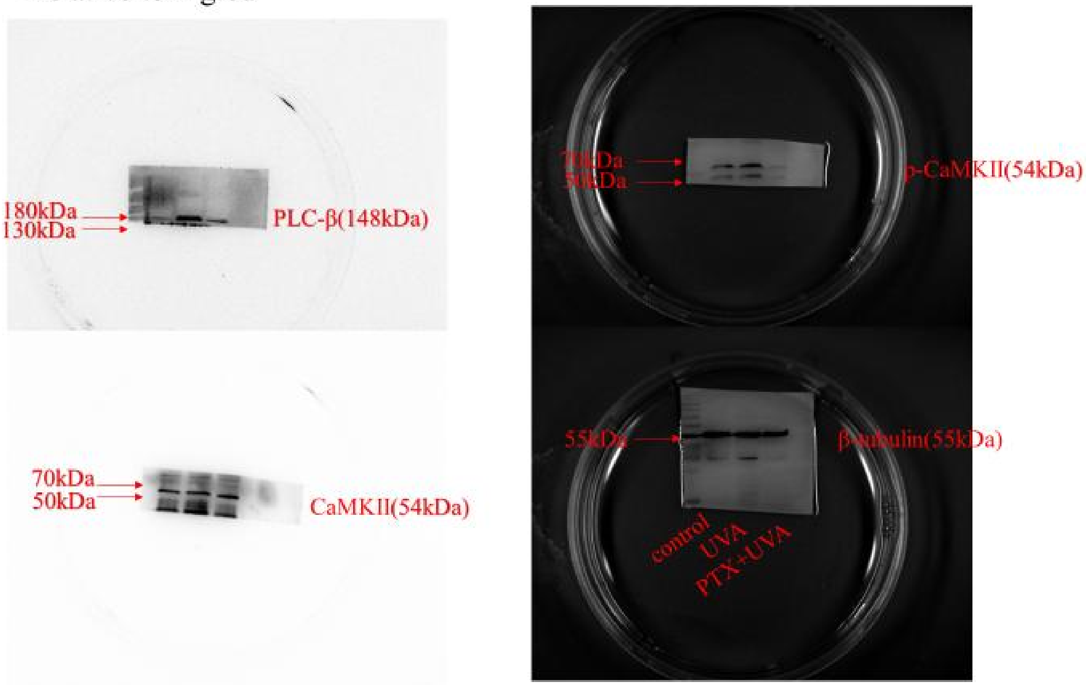

Relative to Fig.6e

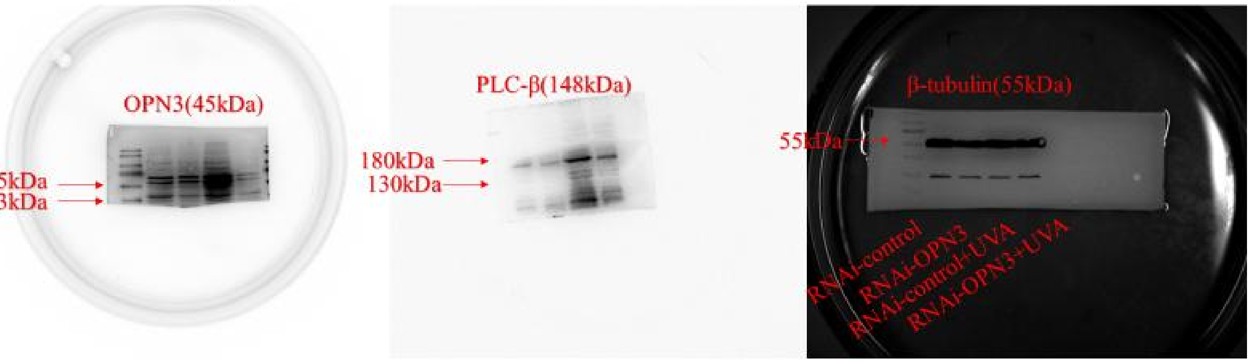

Relative to Fig.6g

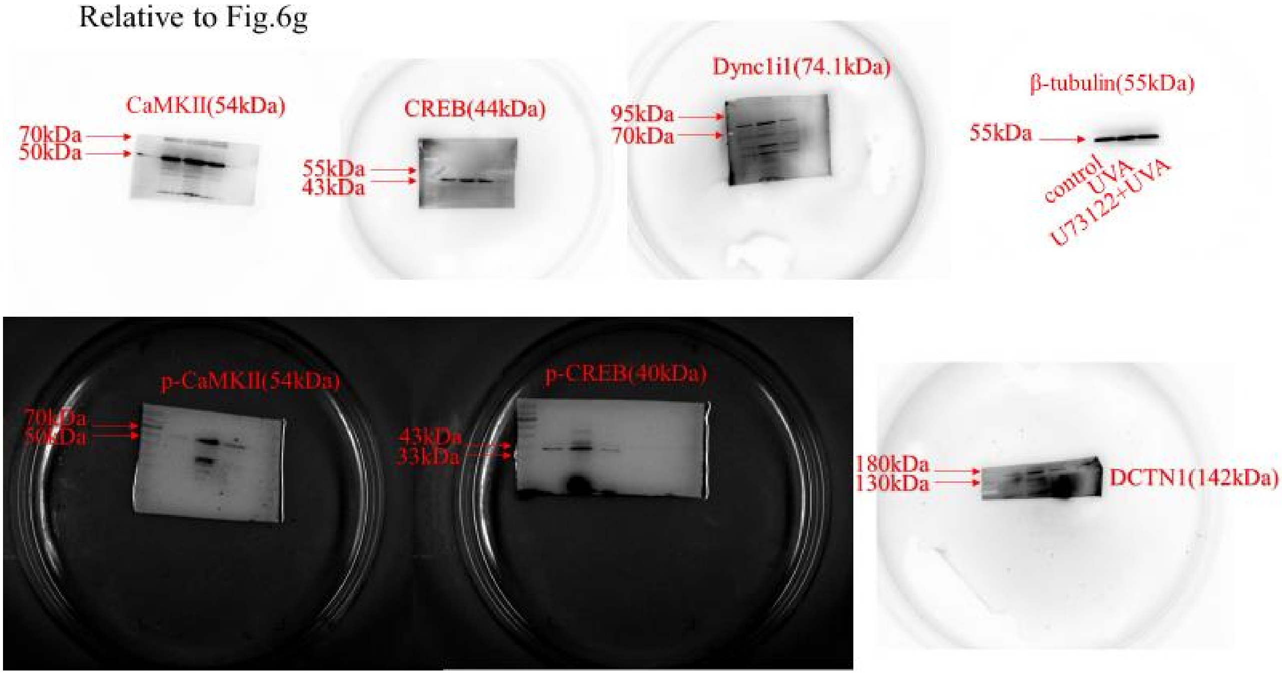

Relative to Fig.7a

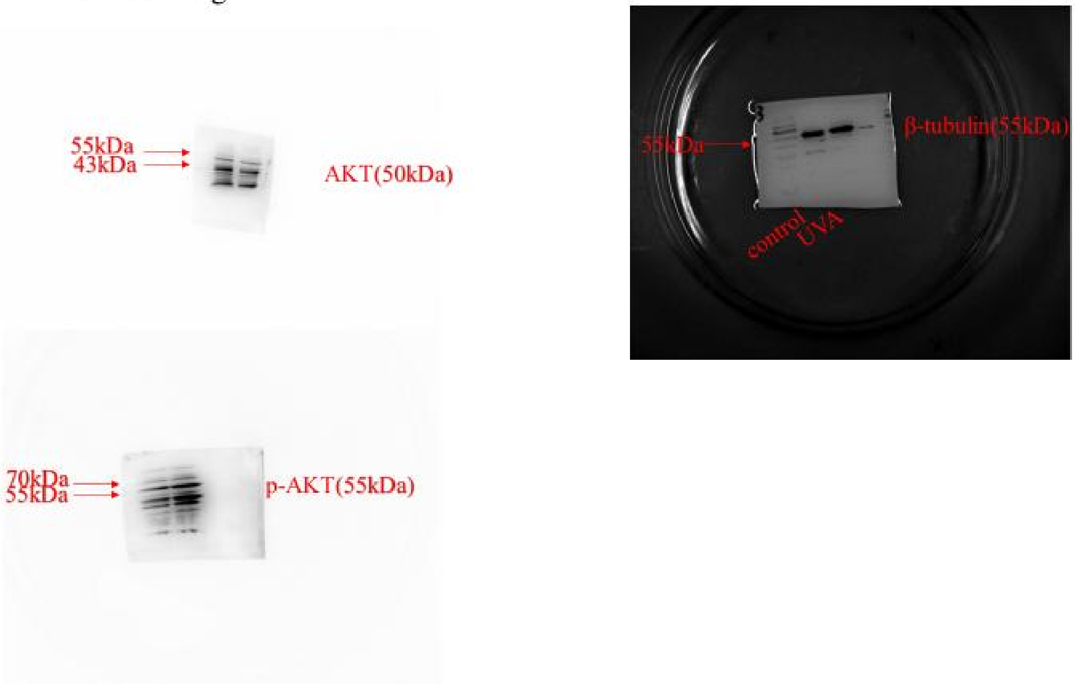

Relative to Fig.7b

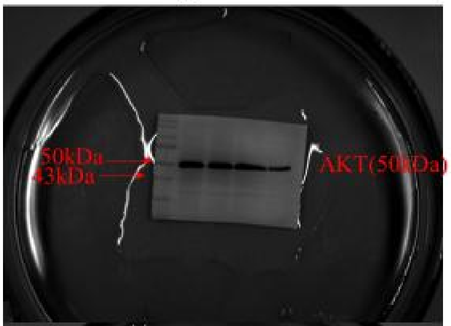

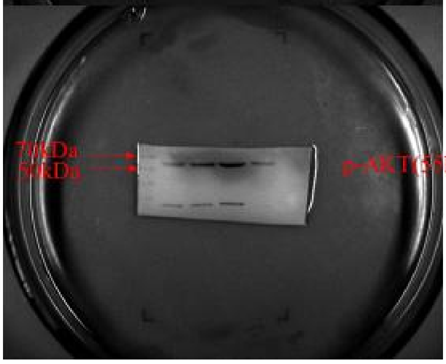

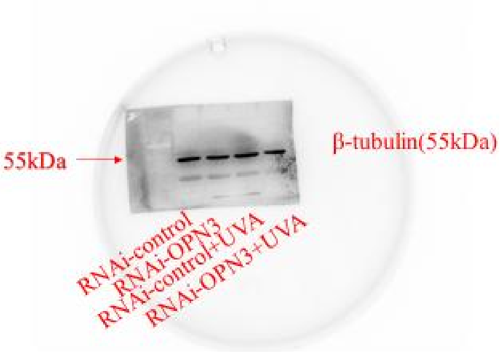

Relative to Fig.7c

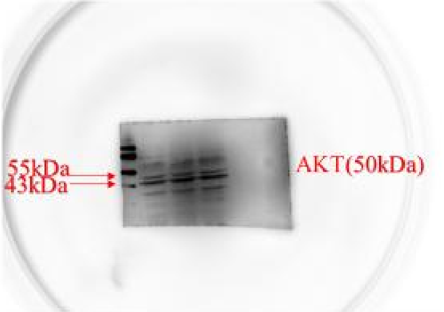

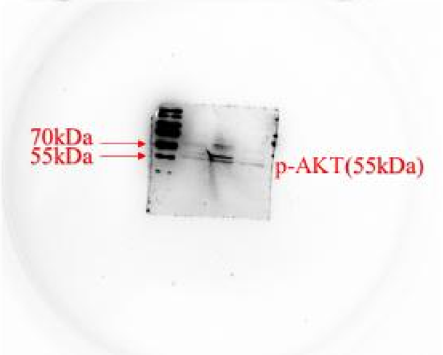

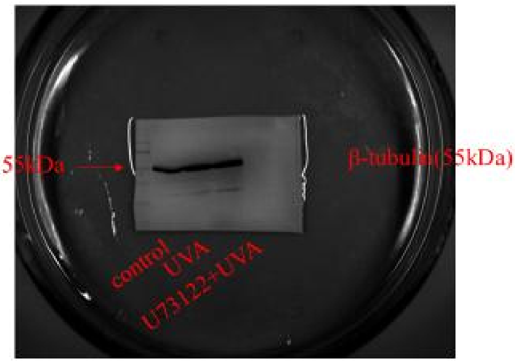

Relative to Fig.7d

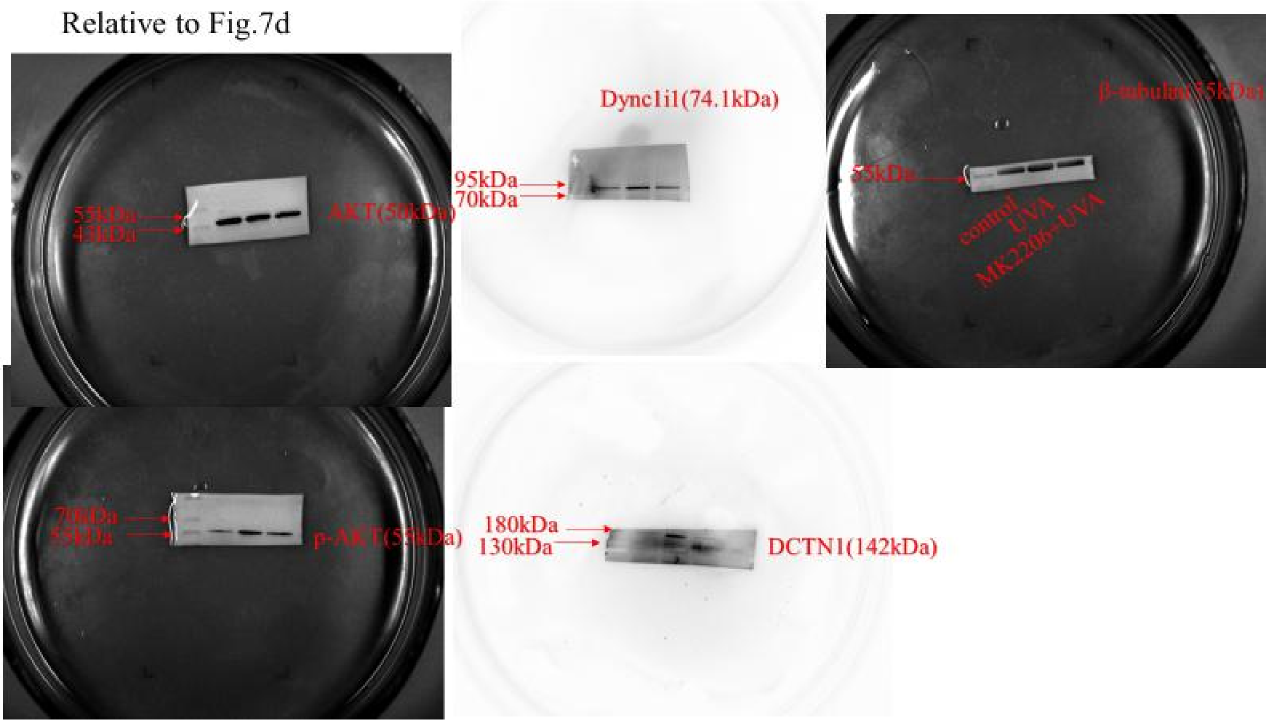

Relative to Fig.s3a

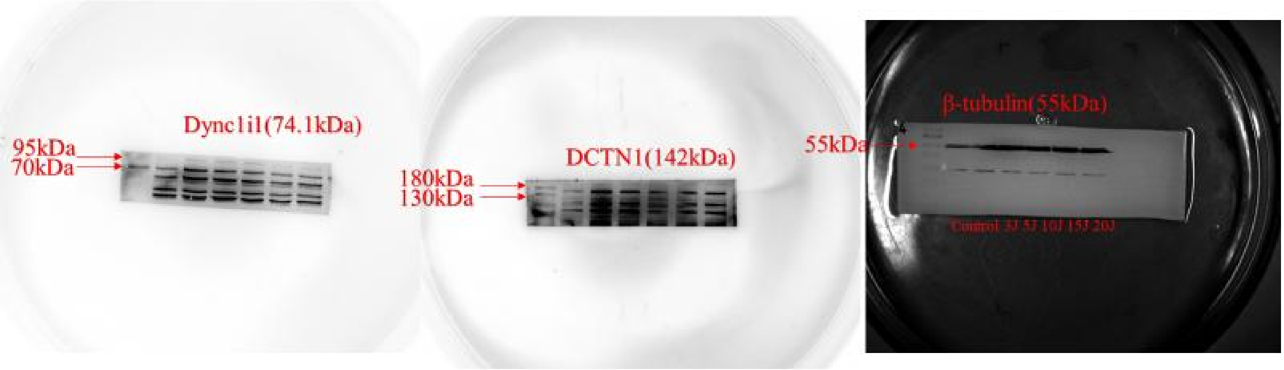

Relative to Fig.s3d

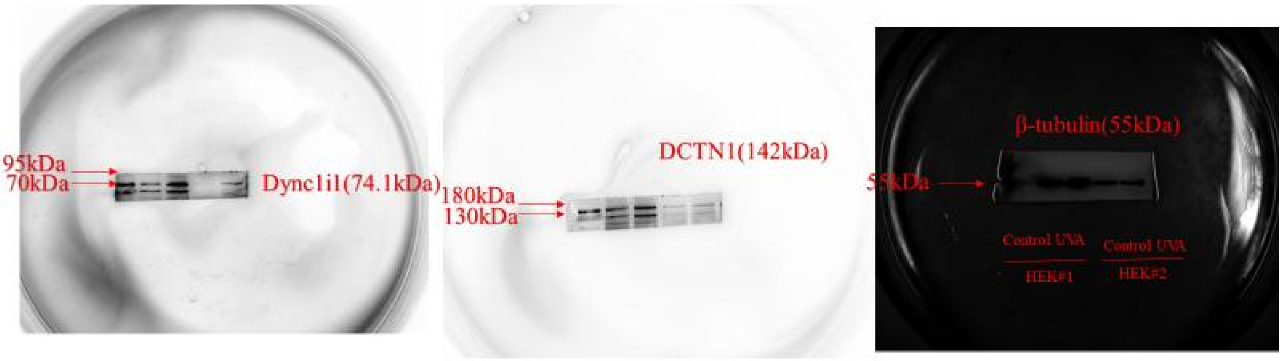

Relative to Fig.s4b

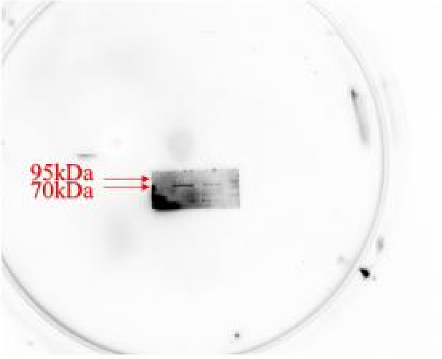

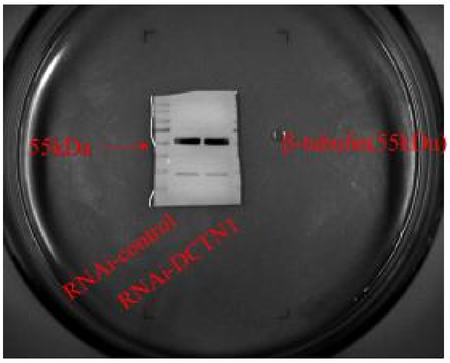

Relative to Fig.s6b

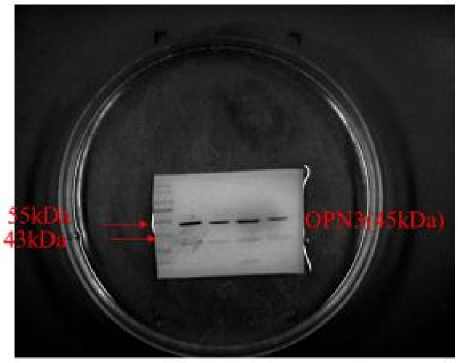

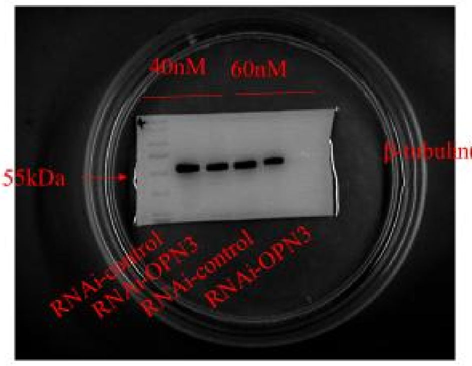

Relative to Fig.s7c

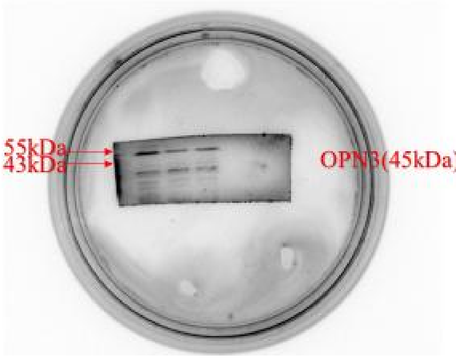

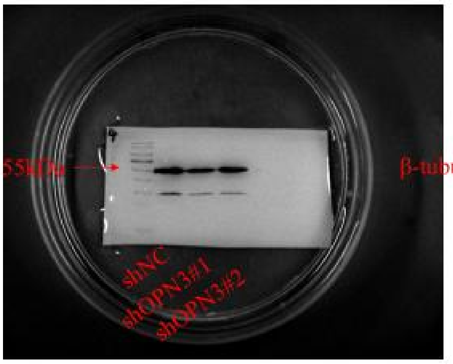

Relative to Fig.s7f

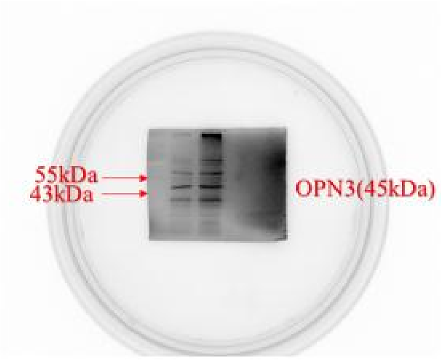

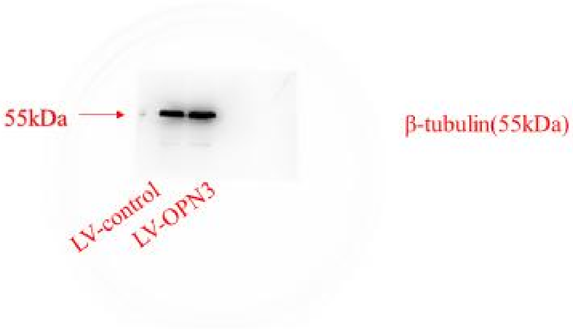

Supplement: Supplementary file 2 — Supplementary Figures [file 42003_2023_4621_MOESM2_ESM.pdf]
