# Supplementary material for: Opsin 3 mediates UVA-induced keratinocyte supranuclear melanin cap formation
Source: Commun Biol. 2023 Mar 3;6:238. doi: 10.1038/s42003-023-04621-8 (PMC9984416; doi:10.1038/s42003-023-04621-8)
Supplement: Supplementary file 3 — Description of Additional Supplementary Files [file 42003_2023_4621_MOESM3_ESM.pdf]

Description of Additional Supplementary Files

File name: Supplementary tables

Description: All source data underlying the graphs and charts.

File name: Supplementary Information

Description: All blots accompanied by size markers in every figure panel.
