# Supplementary material for: Opsin 3 mediates UVA-induced keratinocyte supranuclear melanin cap formation
Source: Commun Biol. 2023 Mar 3;6:238. doi: 10.1038/s42003-023-04621-8 (PMC9984416; doi:10.1038/s42003-023-04621-8)
Supplement: Supplementary file 4 — All source data [file 42003_2023_4621_MOESM4_ESM.pdf]

Figure 2a

opsin expression level on HaCaT

OPN1	OPN2	OPN3	OPN4	OPN5
5.20407E-05	5.65544E-05	0.006848482	2.09891E-05	0.00014071
0.000101235	9.41291E-05	0.019437682	3.90315E-05	5.8752E-05
1.80831E-05	0.000461942	0.017098339	2.65671E-05	0.00010517

Figure 2a

opsin expression level on HEK

OPN1	OPN2	OPN3	OPN4	OPN5
0.000532474	0.000348876	0.00253289	0.000134045	0.00026166
0.000157212	0.000481559	0.001828657	0.000639841	0.00058067
0.000251876	0.000223878	0.001906312	0.000637627	0.00030583

Figure 2f

opsin 3 protein expression level on HaCaT

control	3J	5J	10J	15J
1.629137394	3.987793322	3.014918241	2.805028302	3.06864156
1.094660164	4.583021773	3.989859318	3.395176372	4.36784857
1.965282865	5.842297696	5.340101699	4.854090482	3.76993623

20J
2.344728626
3.151699189
4.768778363

Figure 2g

opsin 3 mRNA expression level on HaCaT

control group	UVA group
0.017873084	0.054972441
0.018783088	0.042690089
0.020327601	0.044033233

Figure 2h

opsin 3 protein expression level on HEK

HEK#1		HEK#2	
control group	UVA group	control group	UVA group
0.592566426	1.002848204	0.642739891	1.225415408
0.671560791	1.119002443	0.685152387	1.212985783
0.637131486	1.382971702	0.788240316	1.797124196

opsin 3 mRNA expression level on HEK

control group	UVA group
0.003412394	0.004230371
0.002421304	0.004597292
0.001694411	0.004230371

Figure 3b

OPN3 protein expression level on HEK

RNAi-control	RNAi-OPN3	RNAi-control+UVA	RNAi-OPN3+UVA
0.877182	0.73766	1.327417	0.442383
1.14485	0.670533	1.405794	0.500658
1.100494	0.658395	1.486807	0.331236

DCTN1 protein expression level on HEK

RNAi-control	RNAi-OPN3	RNAi-control+UVA	RNAi-OPN3+UVA
0.110931939	0.104879884	0.50117824	0.089682457
0.121077476	0.096531232	0.607167177	0.101855322
0.097306144	0.145272356	0.52062984	0.142620772

Dync1i1 protein expression level on HEK

RNAi-control	RNAi-OPN3	RNAi-control+UVA	RNAi-OPN3+UVA
0.190495501	0.192095599	0.70642697	0.22985159
0.211285274	0.227976811	0.817397699	0.219481605
0.136238944	0.212755853	0.744457655	0.229706825

Figure 3c-e

OPN3 mRNA expression level on HEK

RNAi-control	RNAi-OPN3	RNAi-control+UVA	RNAi-OPN3+UVA
0.002096831	0.000169478	0.005622623	0.000234865
0.001759211	0.000147842	0.004523179	0.000338882
0.002399288	0.000158507	0.003139668	0.000269753

DCTN1 mRNA expression level on HEK

RNAi-control	RNAi-OPN3	RNAi-control+UVA	RNAi-OPN3+UVA
0.006311773	0.006668678	0.01717547	0.006317363
0.006052839	0.00828942	0.01061382	0.007279208
0.005294377	0.00827962	0.01717547	0.008785775

Dync1i1mRNA expression level on HEK

RNAi-control	RNAi-OPN3	RNAi-control+UVA	RNAi-OPN3+UVA
0.001478022	0.002142982	0.005819377	0.001106536
0.001811794	0.002294484	0.008639711	0.000820934
0.001397919	0.003612282	0.006769654	0.002257931

Figure 4a

OPN3 protein expression level on HaCaT

shNC	shOPN3	shNC+UVA	shOPN3+UVA
0.54507542	0.377155364	1.102940617	0.433696272
0.72065752	0.34698206	0.984054172	0.357571818
0.734801443	0.385278159	1.193427035	0.396395462

DCTN1 protein expression level on HaCaT

shNC	shOPN3	shNC+UVA	shOPN3+UVA
0.114371379	0.107232692	0.29689144	0.099342567
0.150549001	0.132803905	0.358222744	0.104383806
0.089562573	0.122719486	0.33250664	0.128623702

Dync1i1 protein expression level on HaCaT

shNC	shOPN3	shNC+UVA	shOPN3+UVA
0.184214	0.235837692	0.472476687	0.273315092
0.190147057	0.13413556	0.451623414	0.158085427
0.269338242	0.250679607	0.440571298	0.223779111

Figure 4b-d

OPN3 mRNA expression level on HaCaT

shNC	shOPN3	shNC+UVA	shOPN3+UVA
0.019370433	0.008728806	0.013139006	0.009322935
0.018645871	0.006412044	0.013888167	0.010783729
0.014528202	0.00728932	0.014528202	0.009752582

DCTN1 mRNA expression level on HaCaT

shNC	shOPN3	shNC+UVA	shOPN3+UVA
0.007188966	0.003960779	0.008912217	0.005819053
0.006896117	0.004693891	0.00800435	0.005839255
0.006592311	0.004710187	0.008060025	0.005601388

Dync1i1 mRNA expression level on HaCaT

shNC	shOPN3	shNC+UVA	shOPN3+UVA
0.000147193	4.99208E-05	0.001718065	0.000228582
0.000144665	5.25846E-05	0.001537711	0.000264398
0.000147193	8.99823E-05	0.001315658	0.000210338

Figure 4e

OPN3 protein expression level on HaCaT

LV-control	LV-OPN3	LV-control+UVA	LV-OPN3+UVA
0.170635125	0.325109068	0.553898764	0.763301519
0.163297391	0.335870642	0.548386736	0.752607978

0.147616074	0.32241577	0.46463115	0.788932635

DCTN1 protein expression level on HaCaT

LV-control	LV-OPN3	LV-control+UVA	LV-OPN3+UVA
0.042945286	0.150331006	0.303468344	0.405937907
0.092379447	0.112004423	0.242365785	0.336417489
0.051278624	0.086091365	0.266037606	0.397336365

Dync1i1 protein expression level on HaCaT

LV-control	LV-OPN3	LV-control+UVA	LV-OPN3+UVA
0.11138993	0.136035684	0.215587544	0.285155245
0.141290219	0.151449543	0.230980204	0.334875721
0.157203131	0.159952085	0.234934813	0.28834175

Figure 4f-h

OPN3 mRNA expression level on HaCaT

LV-control	LV-OPN3	LV-control+UVA	LV-OPN3+UVA
0.080214119	0.433769344	0.138740866	0.562529242
0.064928694	0.427797513	0.151118879	0.773782497
0.067451765	0.481297222	0.13742121	0.622005827

DCTN1 mRNA expression level on HaCaT

LV-control	LV-OPN3	LV-control+UVA	LV-OPN3+UVA
0.008820034	0.008974206	0.013649576	0.205897754
0.012090352	0.009618316	0.015843117	0.189464571
0.009194584	0.00857885	0.014528202	0.20877198

Dync1i1 mRNA expression level on HaCaT

LV-control	LV-OPN3	LV-control+UVA	LV-OPN3+UVA
0.007494251	0.007365504	0.03742121	0.275476279
0.007139308	0.008032139	0.024518253	0.270743761
0.007894152	0.007651721	0.025916236	0.26061644

Figure 5a

Calclum-flux-relative units

control group	UVA group
46.32	88.96
51.23	99.17
49.75	94.15

Figure 5b

Calclum-flux-relative units

control group	UVA group
63.45	98.23
49.78	99.17
45.24	91.45

Figure 5c

p-CaMKII protein expression level

control group	UVA group
0.384387919	1.443771579
0.383802564	1.250790408
0.541562594	1.163389513

p-CREB protein expression level

control group	UVA group
0.24893805	0.645916525
0.267175525	0.538185831
0.227613903	0.607793424

Figure 5d

p-CaMKII protein expression level

control group	UVA group
0.372540244	1.296935364
0.444665455	0.791943737
0.481184983	1.480329663

p-CREB protein expression level

control group	UVA group
0.324101443	0.911885619
0.510707281	1.049431851
0.511248081	1.120914499

Figure 5e

Calclum-flux-relative units

RNAi-control	RNAi-OPN3	RNAi-control+UVA	RNAi-OPN3+UVA
75.5	76.34	91.22	70.63
76.44	63.97	90.72	73.08
77.18	57.04	92	62.01

Figure 5f

OPN3 protein expression level

RNAi-control	RNAi-OPN3	RNAi-control+UVA	RNAi-OPN3+UVA
0.400114326	0.284352035	0.967073824	0.181702872
0.406450594	0.276958914	0.813497022	0.156421145
0.343993036	0.227000838	0.65213355	0.10001473

p-CaMKII protein expression level

RNAi-control	RNAi-OPN3	RNAi-control+UVA	RNAi-OPN3+UVA
0.319043907	0.298679144	0.560364787	0.147720693
0.278910292	0.213550878	0.669331693	0.117286502
0.391548938	0.311216852	0.710796531	0.183358421

p-CREB protein expression level

RNAi-control	RNAi-OPN3	RNAi-control+UVA	RNAi-OPN3+UVA
0.19732755	0.16549458	0.455734531	0.127441616
0.184737935	0.163665951	0.456210007	0.122624103
0.157873178	0.190788088	0.41715564	0.115186331

Figure 6a

Calclum-flux-relative units

control	UVA	PTX+UVA
59.13	96.22	44.69
62.45	90.72	34.22
62.01	85.5	53.73

Figure 6b

p-CaMKII protein expression level

control	UVA	PTX+UVA
0.250870879	0.584996453	0.207181564
0.354204106	0.78768696	0.226560007
0.406199637	0.92094698	0.150574369

p-CREB protein expression level

control	UVA	PTX+UVA
0.372831421	1.08242872	0.411931748
0.309301784	1.442209372	0.533524995
0.395891669	1.272440031	0.513676008

Figure 6f

Calclum-flux-relative units

control	UVA	U73122+UVA
20.46	37.26	12.59
18.27	36.26	5.86
18.61	40.84	7.34

Figure 6g

p-CaMKII protein expression level

control	UVA	U73122+UVA
0.259075361	0.573988758	0.273931377
0.246654925	0.622103034	0.259409375
0.263928097	0.556091985	0.257743497

p-CREB protein expression level

control	UVA	U73122+UVA
0.168446322	0.500673488	0.272430309
0.162600668	0.525152635	0.216672479
0.246682304	0.640293036	0.255228287

DCTN1 protein expression level

control	UVA	U73122+UVA
0.168516668	0.525827394	0.202631888
0.209640022	0.531925746	0.20088886
0.167258248	0.495672054	0.192435432

Dync1i1 protein expression level

control	UVA	U73122+UVA
0.301883806	0.726284059	0.183659177
0.28774684	0.541691269	0.129553874
0.3607986	0.590120634	0.147452985

Figure 7a

p-AKT protein expression level

control	UVA
0.306035604	0.644726713
0.354793867	0.835405264
0.338738213	0.779780091

Figure 7b

p-AKT protein expression level

RNAi-control	RNAi-OPN3	RNAi-control+UVA	RNAi-OPN3+UVA
0.526906497	0.730087673	1.463681826	0.615625409
0.640777963	0.724564449	1.766496173	0.586138437
0.730263127	0.624406079	1.423005551	0.917379633

Figure 7c

p-AKT protein expression level

control	UVA	73122+UVA
0.177692305	0.550759957	0.049469584
0.136100838	0.805614971	0.122800825
0.089090551	0.715976752	0.065227322

Figure 7d

p-AKT protein expression level

control	UVA	MK2206+UVA
0.265015599	0.653888043	0.301057036
0.248477819	0.619690208	0.340377611
0.288033845	0.804450858	0.418000584

Dync1i1 protein expression level

control	UVA	MK2206+UVA
0.230142509	0.427487972	0.248412294
0.260862798	0.463168757	0.227883256
0.249243223	0.580715479	0.287857763

DCTN1 protein expression level

control	UVA	MK2206+UVA
0.136411607	0.646081144	0.100368128
0.183247296	0.457411629	0.10972298
0.130033276	0.566466657	0.100286375

Figure. S3a

DCTN1 protein expression level

control	0.17019792	0.25494856	0.177872
3J	0.591892041	0.77483318	0.6681774
5J	0.410046221	0.41067069	0.3508506
10J	0.28927148	0.2992706	0.3613991
15J	0.308633623	0.36322751	0.3182048
20J	0.328373946	0.41249138	0.3772307

Dync1i1 protein expression level

control	0.325517715	0.32793464	0.3605618
3J	0.665233127	0.72933604	0.6051627
5J	0.288702885	0.28194799	0.2922402
10J	0.267327336	0.31040817	0.2550811
15J	0.140448187	0.2133019	0.1956864
20J	0.21647294	0.27902731	0.2297808

Figure. S3b

Dync1i1 mRNA expression level	control group	UVA group
	0.000924	0.003683
	0.0000938	0.005031
	0.0000726	0.008851

Figure. S3c

DCTN1 mRNA expression level	control group	UVA group
	0.024518	0.42986
	0.06754	0.712025
	0.04944	1.21841

Figure. S3d

DCTN1 protein expression level

HEK#1		HEK#2	
control group	UVA group	control [illegible]	UVA group
0.345627623	0.55458004	0.28878	0.5925522
0.402991365	0.554191812	0.2003901	0.5098766
0.354216563	0.672111708	0.1782868	0.6780705

Dync1i1Dync1i1 protein expression level

HEK#1		HEK#2	
control group	UVA group	control group	UVA group
0.360395516	1.262958099	0.1712175	1.3336242
0.360395516	1.143078528	0.1555096	1.4376675
0.376846857	0.967149318	0.2233728	1.3802083

Figure. S3e,f

DCTN1 mRNA expression level

control group	UVA group
0.000844275	0.003594483
0.000809882	0.00596195
0.000673983	0.004693891

Dync1i1 mRNA expression level

control group	UVA group
0.000498541	0.000973184
0.000481559	0.001506065
0.000539908	0.001223305

Figure. S4a

DCTN1 mRNA expression level

control	siDCTN1#1	siDCTN1#2	siDCTN1#3	siDCTN1#4
0.0051301	0.0017534	0.0047458	0.0020439	0.0030207
0.0051951	0.0018414	0.0052603	0.0027719	0.0040407
0.0056057	0.0014455	0.0046582	0.0022061	0.003519

Figure. S6a

OPN3 mRNA expression level

40nM

RNA-control	RNAi-OPN3
0.001357342	0.000697751
0.001141384	0.000514336
0.00086801	0.000534323

60nM

RNA-control	RNAi-OPN3
0.008850655	0.001980232
0.013461659	0.002520269
0.010934265	0.002850655

Figure. S6b

OPN3 mRNA expression level

40nM

RNAi-control	RNAi-OPN3
0.583947571	0.418845995
0.608376711	0.434732039
0.423118687	0.24857434

60nM

RNAi-control	RNAi-OPN3
0.527014095	0.233409869
0.62892198	0.265099398
0.514945459	0.264452759

Figure. S7b

OPN3 mRNA expression level

shNC	shOPN3#1	shOPN3#2
0.039968	0.006003	0.004743
0.03396	0.005505	0.002251
0.040808	0.002879	0.00324

Figure. S7c

OPN3 protein expression level

shNC	shOPN3#1	shOPN3#2
0.570493097	0.2034136	0.216796
0.561618021	0.213686	0.2357105
0.477493011	0.2213162	0.2346296

Figure. S7e

OPN3 mRNA expression level

LV-control	LV-OPN3
0.010525	0.111796
0.014279	0.136313
0.011359	0.159873

Figure. S7f

OPN3 protein expression level

LV-control	LV-OPN3
0.284225426	0.4797914
0.171761449	0.390727
0.125309007	0.4030247

Fig. s9a

PTX

control	1	1	1
100ng	0.95253	1.042336	0.944689
150ng	1.035511	1.05103	1.041713
200ng	1.015639	0.969382	1.018743
250ng	0.790616	0.800605	0.796582
300ng	0.666973	0.586657	0.632488
350ng	0.390616	0.349083	0.336457

Fig. s9b

U73122

control	1	1	1
4.5µM	1.001904	0.96127	1.075061
9µM	0.925444	0.936019	0.953393
13.5µM	0.78236	0.760496	0.759771
18µM	0.653553	0.624322	0.672772
22.5µM	0.449556	0.489543	0.552903
27µM	0.292989	0.10395	0.297138

Fig. s9c

MK2206

control	1	1	1
3µM	1.062672	1.036915	1.06583
6µM	0.963269	0.990595	1.047596
9µM	1.006657	1.035034	1.046886
12µM	0.967401	1.019751	0.943405
15µM	0.703168	0.901246	0.881838
18µM	0.471074	0.558664	0.673928
21µM	0.251377	0.338349	0.305707
24µM	0.097567	0.124853	0.120294
