# Supplementary material for: Opsin 3 mediates UVA-induced keratinocyte supranuclear melanin cap formation
Source: Commun Biol. 2023 Mar 3;6:238. doi: 10.1038/s42003-023-04621-8 (PMC9984416; doi:10.1038/s42003-023-04621-8)
Supplement: Supplementary file 5 — reporting summary [file 42003_2023_4621_MOESM5_ESM.pdf]

nature portfolio

Corresponding author(s): Hongguang Lu

Last updated by author(s): Feb 5, 2023

Reporting Summary

Nature Portfolio wishes to improve the reproducibility of the work that we publish. This form provides structure for consistency and transparency in reporting. For further information on Nature Portfolio policies, see our Editorial Policies and the Editorial Policy Checklist.

Statistics

For all statistical analyses, confirm that the following items are present in the figure legend, table legend, main text, or Methods section.

n/a	Confirmed	
☒	☐	The exact sample size (*n*) for each experimental group/condition, given as a discrete number and unit of measurement
☐	☒	A statement on whether measurements were taken from distinct samples or whether the same sample was measured repeatedly
☒	☐	The statistical test(s) used AND whether they are one- or two-sided<br>*Only common tests should be described solely by name; describe more complex techniques in the Methods section.*
☒	☐	A description of all covariates tested
☒	☐	A description of any assumptions or corrections, such as tests of normality and adjustment for multiple comparisons
☐	☒	A full description of the statistical parameters including central tendency (e.g. means) or other basic estimates (e.g. regression coefficient) AND variation (e.g. standard deviation) or associated estimates of uncertainty (e.g. confidence intervals)
☒	☐	For null hypothesis testing, the test statistic (e.g. F, t, r) with confidence intervals, effect sizes, degrees of freedom and P value noted<br>*Give P values as exact values whenever suitable.*
☒	☐	For Bayesian analysis, information on the choice of priors and Markov chain Monte Carlo settings
☐	☒	For hierarchical and complex designs, identification of the appropriate level for tests and full reporting of outcomes
☒	☐	Estimates of effect sizes (e.g. Cohen's *d*, Pearson's *r*), indicating how they were calculated

Our web collection on statistics for biologists contains articles on many of the points above.

Software and code

Policy information about availability of computer code

Data collection	lease see the Materials and methods
Data analysis	The data were presented as mean ± SEM and were based on at least three separate experiments. Statistical significance was determined by t-test (and nonparametric tests).For three or more comparisons the ANOVA with post-test used. Differences were considered significant when the P value < 0.05. All analyses were carried out with GraphPad Prism Version 8.0 software (GraphPad Software, San Diego, CA, USA).

For manuscripts utilizing custom algorithms or software that are central to the research but not yet described in published literature, software must be made available to editors and reviewers. We strongly encourage code deposition in a community repository (e.g. GitHub). See the Nature Portfolio guidelines for submitting code & software for further information.

Data

Policy information about availability of data

All manuscripts must include a data availability statement. This statement should provide the following information, where applicable:

- Accession codes, unique identifiers, or web links for publicly available datasets
- A description of any restrictions on data availability
- For clinical datasets or third party data, please ensure that the statement adheres to our policy

Please log in the (https://doi.org/10.5061/dryad.wh70rxwrx) to view all the original data of the manuscript.

Human research participants

Policy information about studies involving human research participants and Sex and Gender in Research.

Reporting on sex and gender	This study did not involve sex and gender.
Population characteristics	NA
Recruitment	NA
Ethics oversight	NA

Note that full information on the approval of the study protocol must also be provided in the manuscript.

Field-specific reporting

Please select the one below that is the best fit for your research. If you are not sure, read the appropriate sections before making your selection.

☒ Life sciences ☐ Behavioural & social sciences ☐ Ecological, evolutionary & environmental sciences

For a reference copy of the document with all sections, see nature.com/documents/nr-reporting-summary-flat.pdf

Life sciences study design

All studies must disclose on these points even when the disclosure is negative.

Sample size	The sample size calculation is not applicable to this study.
Data exclusions	Not applicable
Replication	3 or more
Randomization	Not relevant
Blinding	Not relevant-any images or data were presented in full.

Reporting for specific materials, systems and methods

We require information from authors about some types of materials, experimental systems and methods used in many studies. Here, indicate whether each material, system or method listed is relevant to your study. If you are not sure if a list item applies to your research, read the appropriate section before selecting a response.

Materials & experimental systems

n/a	Involved in the study	
☐	☒	Antibodies
☐	☒	Eukaryotic cell lines
☒	☐	Palaeontology and archaeology
☒	☐	Animals and other organisms
☒	☐	Clinical data
☒	☐	Dual use research of concern

Methods

n/a	Involved in the study	
☒	☐	ChIP-seq
☐	☒	Flow cytometry
☒	☐	MRI-based neuroimaging

Antibodies

Antibodies used

kin tissue immunofluorescence:
an anti-OPN3 rabbit monoclonal antibody (ab228748; Abcam) conjugated to Alexa Fluor 488-labelled goat anti-rabbit IgG (A0423; Beyotime); anti-pancytokeratin mouse monoclonal antibody (sc-81703; Santa Cruz Biotechnology, Inc.) conjugated to Cy3-labelled goat anti-mouse IgG (A0521; Beyotime); anti-Dync1i1 rabbit monoclonal antibody (AB_2846296, Affinity Biosciences, Ltd.) conjugated to an Alexa Fluor 488-labelled goat anti-rabbit IgG (A0423; Beyotime); and anti-DCN1 rabbit monoclonal antibody (AB_2838577, Affinity Biosciences, Ltd.) conjugated to Alexa Fluor 488-labelled goat anti-rabbit IgG (A0423; Beyotime).
Cell immunofluorescence:
rabbit anti-OPN3 monoclonal antibody (cat. no. AB_2837240, 1:50, Affinity Biosciences Ltd., Beijing, China) and a mouse anti-

	pancytokeratin antibody (cat. no. sc-81703, 1:50, Santa Cruz Biotechnology, Inc., Shanghai, China) .<br>Western blot (WB) analysis:<br>anti-OPN3 (1:1000, cat. no. AB_2837240, Affinity Biosciences Ltd., Beijing, China), anti-DCTN1 (1:1000, AB_2838577, Affinity Biosciences Ltd., Beijing, China), anti-cytoplasmic dynein 1 intermediate chain 1 (Dync1i1, 1:1000, AB_2846296, Affinity Biosciences LTD, Beijing, China), anti- calmodulin-dependent protein kinase II (CaMKII, 1:1000, ab126789, Abcam, Cambridge, UK), anti-phospho-(p)-CaMKII (1:1000, ab124880, Cambridge, UK), anti-CREB (1:1000, ab32515, Abcam, Cambridge, UK), anti-p-CREB (phosphorylated at S133) (1:1000, ab220798, Abcam, Cambridge, UK), anti-Akt (1:1000, ABP0059, Abbkine Scientific, Wuhan, China), anti-p-Akt (phosphorylated at Ser473) (1:1000, ABP0030, Abbkine Scientific, Wuhan, Wuhan, China) and anti-beta-tubulin (1:1000, AB_2827688, Affinity Biosciences Ltd., Beijing, China). secondary antibody (goat anti-rabbit IgG H&L, ab97051, Abcam, or goat anti-mouse IgG H&L, ab6789, Abcam)
Validation	All the primary antibodies and secondary antibody were previously validation by commercial providers and previously described in literature.

Eukaryotic cell lines

Policy information about cell lines and Sex and Gender in Research

Cell line source(s)	Human immortalized keratinocytes (HaCaT) were purchased from Kunming Cell Bank of Type Culture Collection, Chinese Academy of Science (KCB200442YJ, Kunming, China).
Authentication	The cell lines uesd were authenticated
Mycoplasma contamination	Cell lines were tested for Mycoplasma contamination
Commonly misidentified lines<br>(See ICLAC register)	N/A

Flow Cytometry

Plots

Confirm that:

☒ The axis labels state the marker and fluorochrome used (e.g. CD4-FITC).

☒ The axis scales are clearly visible. Include numbers along axes only for bottom left plot of group (a 'group' is an analysis of identical markers).

☒ All plots are contour plots with outliers or pseudocolor plots.

☒ A numerical value for number of cells or percentage (with statistics) is provided.

Methodology

Sample preparation	Described in detail in method section.
Instrument	Flow cytometry analysis was performed with a BD LSRIIF(BD Biosciences, San Jose, CA, USA)
Software	BD FACSDiva,FlowJo software 10.6.1 (BD)
Cell population abundance	see manuscript
Gating strategy	see details in method section with links to figures showing gating strategy(original data).

☐ Tick this box to confirm that a figure exemplifying the gating strategy is provided in the Supplementary Information.
